# Indications and contraindications of dental implants in medically compromised patients: Update

**DOI:** 10.4317/medoral.19565

**Published:** 2014-03-08

**Authors:** Rafael Gómez-de Diego, María del Rocío Mang-de la Rosa, María J. Romero-Pérez, Antonio Cutando-Soriano, Antonio López-Valverde-Centeno

**Affiliations:** 1 Department of Stomatology, University Rey Juan Carlos, Madrid, Spain; 2 Department of Special Care patients, School of Dentistry, University of Granada, Spain; 3 Department of Special Care patients, School of Dentistry, University of Granada, Granada, Spain; 4 Special Care Professor, University of Granada, Department of Special Care Dentistry, School of Dentistry, University of Granada, Granada, Spain; 5 Department of Surgery, School of Dentistry, Faculty of Medicine, University of Salamanca, Salamanca, Spain

## Abstract

The aim of this study was to review the current scientific literature in order to analyse the indications and contraindications of dental implants in medically compromised patients. A reference research was carried out on PubMed using the key words “implant” AND (oral OR dental) AND (systemic disease OR medically compromised), in articles published between 1993 and 2013. The inclusion criteria were the following: clinical studies in which, at least, 10 patients were treated, consensus articles, reviewed articles and meta-analysis performed in humans treated with dental implants, and which included the disease diagnosis. A total of 64 articles were found, from which 16 met the inclusion criteria. Cardiac systemic diseases, diabetic endocrine pathologies or controlled metabolic disorders do not seem to be a total or partial contraindication to the placement of dental implants. Tobacco addiction, and head and neck radiotherapy are correlated to a higher loss of dental implants. Patients suffering from osteoporosis undergoing biphosphonates therapy show an increased risk of developing bone necrosis after an oral surgery, especially if the drugs are administered intravenously or they are associated to certain concomitant medication.

** Key words:**Dental implants, medically compromised patient, systemic diseases.

## Introduction

A medically compromised patient (MCP) can be described, as the one who has a distinctive physical or mental feature regarding the people of the same age. In this sort of patients there is a higher risk of interactions between their disease and the implant surgery, implying a higher medical risk. This group need, therefore, to fill in a medical questionnaire and to undergo a previous exhaustive medical examination, which will help not only to determine the specific measures that must be adopted (1), but also to carry out the estimation of the patient’s risk. The system proposed by the American Society of Anesthesiologists in 1941, and the one adapted by McCarthy and Malamed (2,3) to the dental patient were used to define the patient’s risk. These classifications as well as the medical history allow us to identify the systemic disease and the success rate expected in the MCP that is going to be rehabilitated with dental implants. It seems like the medical control of the disease is more important than the disease itself. This evidences the need of carrying out personalized medical examinations (4).

Medical advances have made possible the increase of the survival rate of certain types of medically compromised patients, increasing thus the prevalence of MCP who request the rehabilitation of their total or partially edentulous maxillary bones with dental implants. This is due to the high success rate of this surgical technique and its benefits to the patients’ function and quality of life.

Among scientific literature there is an enormous variety of studies that analyse the most common systemic diseases presented by patients undergoing dental treatment, correlating it with adequate and safe clinical practices and existing little information which associate these diseases with dental implants surgery.

## Objectives

The aim of this study is to thoroughly revise the current literature, in order to analyse the indications and contraindications of treating MCP with dental implants.

## Material and Methods

A reference research was carried out at the access portal PubMed, using the keyword “implant*AND (oral OR dental) AND (systemic disease OR medically compromised), limiting the research to articles published in dental journals between 1993 and 2013. Moreover, the articles should be written in English and the abstracts should also be published in that database.

The inclusion criteria were the following: clinical studies in which, at least, 10 patients were treated, consensus articles, review articles and meta-analysis performed in humans treated with dental implants and including the disease diagnosis. The following features were registered for each study: publication year, systemic disease, number of dental implants placed and their survival rate. The goal was to evaluate whether or not exists correlation between: head and neck radiotherapy treatment, intake of biphosphonates, systemic diseases including cardiac systemic diseases, diabetic endocrine pathologies, osteoporosis and tobacco consumption and the lack of osseointegration of the dental implants.

## Results and Discussion

A total of 64 articles were found using the research strategy described above and 18 of them met the inclusion criteria. The latter followed an specific analysis ( Table 1).

**Table 1:**
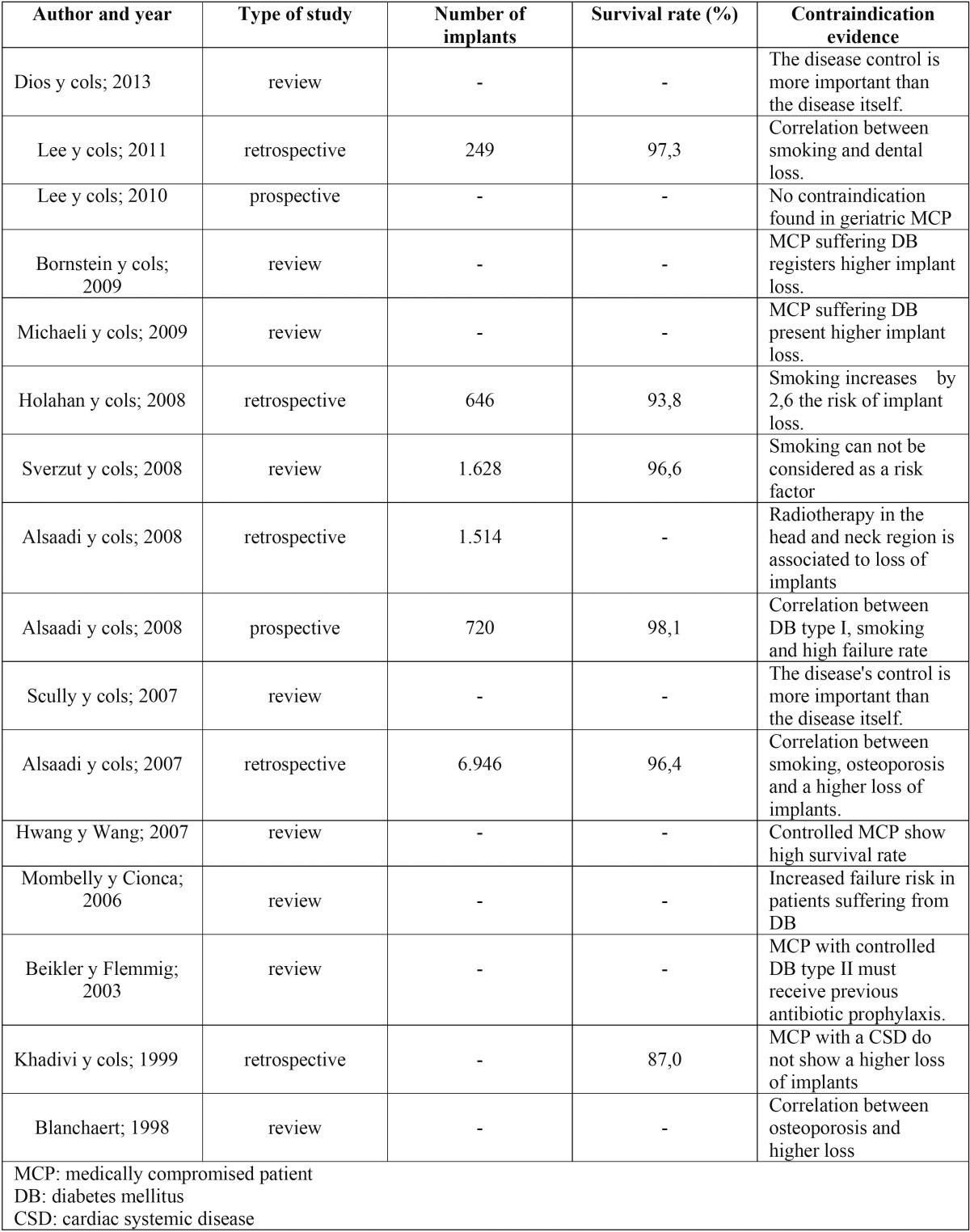
Data collected from the articles that met the inclusion criteria.

The evidence level of implant failures in MCP is limited (5) due to the short number of controlled randomised studies. There are even retrospective studies, with 3 years of follow up, in geriatric MCP (70 years and over), which conclude that controlled systemic diseases should not be considered as a risk factor for dental implants failure (*p*=0,484) subjected to prosthetic charge (6).

The cardiac systemic disease (CSD) can endanger and reduce the amount of oxygen and nutrients in the osseous tissue, which may affect the osseointegration process of dental implants. Some authors (7,8) even point out the relative contraindication of placing dental implants in patients with certain CSD due to their higher risk of developing infective endocarditis. On the contrary, it does not seem to exist correlation between the lack of osseointegration of dental implants and patients with certain CSD, as concluded by Khadivi and cols in their retrospective case study of MCP (n=148) and healthy controlled patients (n=98). There were found 39 patients affected by a cardiac pathology (23,9%), registering a 13% failure rate in these patients and a 12% failure rate in the control group (9).

In none of the studies, except for one, radiotherapy was mentioned as a risk factor associated to the frequency of dental implants loss (10). The authors describe a sample of 1514 implants analysed in 700 patients, with a retrospective two-year follow-up.

This kind of treatment involving ionizing radiation and when placed over the oral cavity, can be relevant in order to explain the association between radiotherapy and loss of dental implants. It has been suggested that therapy with hyperbaric oxygen could reduce the incidence of loss of dental implants in irradiated patients. In a recent systematic review (11), the authors were only able to find a controlled and randomised study, in which dental implants where placed in a group of premedicated patients, compared to another study that used both premedication and hiperbaric therapy, obtaining a 85,2 % survival rate in the first study and a 93,3% survival rate in the second one. This leads the authors to conclude that the use of hyperbaric treatment in patients undergoing implant treatment does not seem to provide significant benefits.

Radiotherapy could be responsible in the reduction of the success rate of dental implants when it is administered in doses exceeding 50 Gy, as it has already been proved for extraoral implants. To that effect, Verdonck and cols performed a case-control study using the maxilla and mandible of six adult Göttingen minipigs. The maxilla and mandible of three minipigs received irradiation exposures at a total dose of 24 Gy and 120 implants were placed with perioperative and postoperative recordings for the implant stability quotient (ISQ) at 8, 16, and 24 weeks after the implant placement. ISQ values recorded immediately after implant placement showed no difference between irradiated and non-irradiated minipigs, but the repeated measurements at the four recording moments showed a decrease of ISQ values when compared with non-irradiated bone (12).

The consumption of tobacco seems to be a factor associated with the increase in the loss of dental implants; Wilson and Nunn established a failure rate 2.5 times higher in patients who smoke (13), and this rate has augmented in recent studies (14,15) up to a 2.6 in smokers as compared with non-smokers. These results seem contradictory with those found by Alsaadi and col in their two years retrospective study, as they concluded that the consumption of tobacco is not a decisive factor in the loss of dental implants (10), which is similar to the results found by Sverzut and col in their retrospective study (16). Among the studies reviewed specifically for this article, 4 of them associated the consumption of tobacco with the implant loss significantly, as opposed to 2 of them already mentioned(8,17-19) ( Table 2).

**Table 2:**
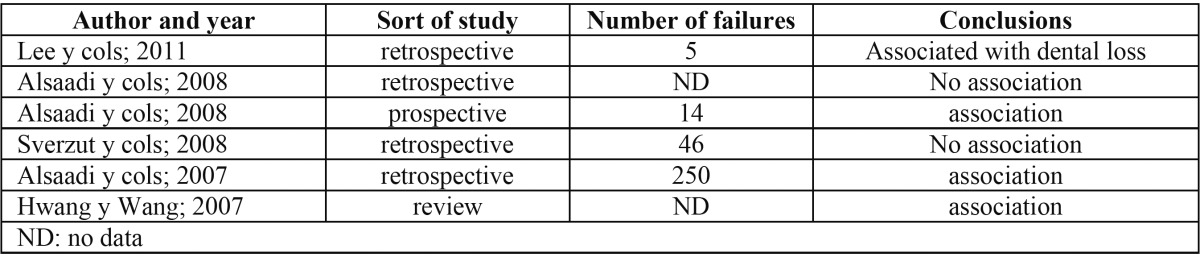
Correlation between snuff consumption and loss of dental implants.

Metabolic changes produced by diabetes are associated with the synthesis of the osteoblastic matrix induced by insulin. The variation in the differentiation of osteoblastic cells and hormones which regulate the calcium metabolism, produce, in the mineral bone tissue homeostasis, an alteration in the level of bone matrix required to produce mature osteocytes which boost the osseointegration of dental implants. Epidemiological case-control studies carried out in animals show a variation in the bone density surrounding the implant in samples of non-controlled diabetic patients (20,21). Most studies reviewed confirm these experimental results. Morris and col. in their 3 year restrospective study, show a higher frequency of implant failure in diabetic patients (7.8%) as compared with healthy patients (6.8%) (22). These data are confirmed in the thorough review of Mombelly and Cionca (23) or in a recent one carried out by Bornstein and col (7). The most recent publications arise different results in spite of insisting on the higher risk of failure in diabetic patients (24). In that way, Alsaadi and col (10) analyzed seven systemic diseases in a restrospective study with a large sample (n=700) and concluded that diabetic endocrine pathology is not associated with a higher frequency of failure in dental implants, though the same author pointed out in another study carried out in the University of Leuven (16) with a smaller sample of patients (n=273) and twelve systemic diseases analyzed, that patients who suffer from diabetes type I have more tendency towards dental implant failure. These results are conflicting with those published by the same author in 2007 (20), when 6946 dental implants were placed in 2004 patients with the loss of 252 implants; the analysis carried out about the early loss of implants points out just one patient who suffered from diabetes type I and the frequency of 4% in early implant failure (n=14) associated with diabetes type II. In the light of the results exposed, the total contraindication of placing dental implants in diabetic patients because of the higher frequency of failure in the osseointegration and risk of infection (24) proposed in the past, has been modified owing to the fact that the risk of augmenting the failure rate in controlled diabetic patients who have been placed dental implants is only relative when they have received an antibiotic prophylaxis protocol and aseptic techniques with chlorhexidine gluconate 0.12% (1,22).

Osteoporosis, metabolic disease which modifies the bone mass and density, is the most frequent bone disorder, which affects sponge bone mainly and is more common in postmenopausal women. It has been considered for a long time that this disease complicates the initial stability of dental implants because of the loss in the sponge bone mass (25). However, current publications show high survival rates, between 93.8 and 100% (17). A recent retrospective study analyzes 646 dental implants placed in 50 or more year old women (n=192), diagnosed with osteopenia or osteoporosis and who were tested their mineral bone density, being the survival rate of the implants 5 years later of 93.8%, which demonstrates the absence of an statistically significant association between this disease and the failure in implants (15).

It has also been observed an adequate percentage of contact bone-implant in women who suffer from osteoporosis through histomorphometry studies. In that way, Melo and col described a case report of a 68 year old postmenopausal woman who had a 62.51% contact bone-implant after 6 years of loading it (26). A recent case-control epidemiological study evaluated a sample of 21 patients, 7 of them were postmenopausal women suffering from osteoporosis and 14 did not show signs of this pathology. The percentage of bone-implant contact was of 46 in the first group as opposed to the 47.84% of the control group (27). Both articles concluded that osteoporosis may not be a contraindication for the placement of dental implants.

The bifosfonates (BF), drugs indicated in the prevention and treatment of illnesses associated to bony resorption (ederly osteoporosis , induced by corticoids, or Paget disease), bony metastasis of cancer from suckles and prostate, syndromes paraneoplásics (wicked hypercalcemia) and multiple myeloma. They can be used for via oral or intravenous.

The PMC in treatment with BF, especially those administered for intravenous via, they present bigger incidence of risk of suffering osteonecrosis (OQN) (BCN, Bone Chemical Necrosis) at the maxillary to the subjected being to oral surgical treatments (28). This way, Kasai and cols., they compare the failure of the dental implants in two groups of women: prescribed with oral BF (n=11) (OBF, Oral Biphosphonates) in front of not prescribed (n=54), obtaining a rate of survival in you implant them of 86% in the cases in front of 95% of the controls (29). There are not unanimous consent, neither conclusive data in the attitude to take before the insert the dental implant in PMC in treatment with oral BF, although the most recent clinical studies show a relationship between the treatment with oral BF, use of dental implant, and the frequency of success and the presence of OQN ( Table 3) they seem to endorse the security of the technique in this type of PMC (30-37). By the other side, it is necessary to make the PMC tried with intravenous BF of the drop periimplantitis incidence, but high risk of OQN . Risk that is increased if the case of PMC receiving treatment with ciclosporin, azathioprine or similar, corticoids or hormonal therapy, in this case its is an absolute contraindication (38-40).

**Table 3:**
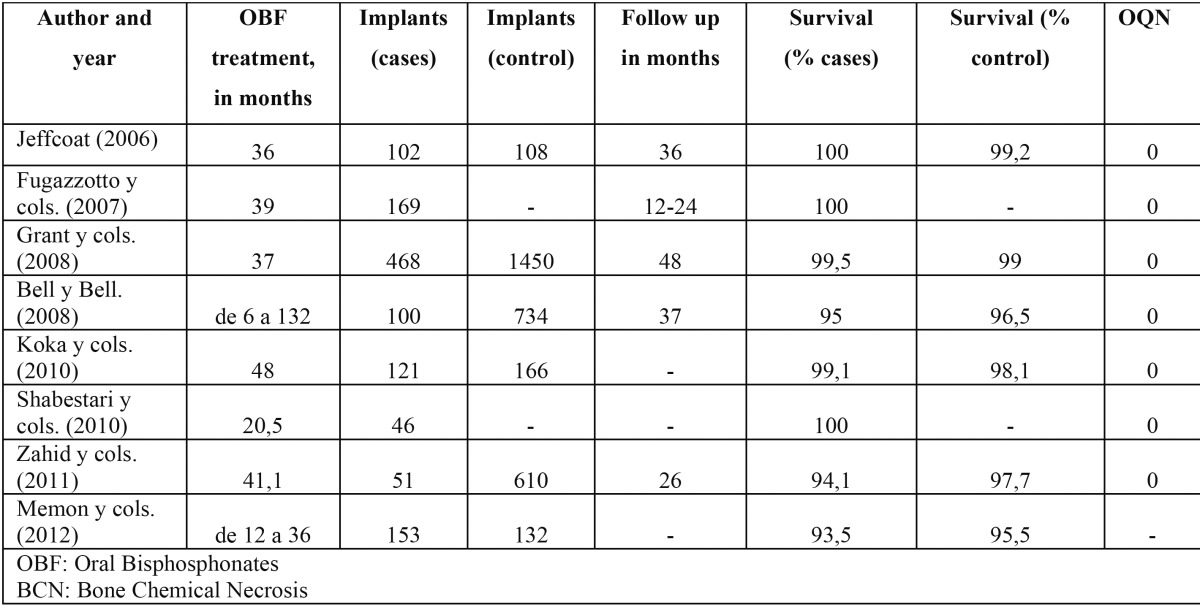
Correlation between consumption of OBF, dental implants and BCN.

## Conclusions

The survival rate of dental implants placed in MCP who suffer from controlled systemic diseases or smoke, does not indicate a total or partial contraindication for the placement of dental implants, as the level of evidence associated with the implant loss is low, it seems to be a secure procedure which do not have to be considered risky, though there is not available information recorded in patients suffering from severe diseases. The consumption of oral biphosphonates by patients who suffer from osteoporosis seems to be a partial contraindication for the treatment with dental implants and the patient must understand the necessity of a longer follow-up period so as to detect any sign of BCN. On the contrary, those patients who have been subjected to radiotherapy protocols in the head or neck region, with doses higher than 50Gy, seem to show lower levels of osseointegration throughout the time, being contraindicated their placement in those patients who have received a therapy with biphosphonates intravenously and when they are associated with hormonal therapy, corticosteroids or immunosupressors.
